# Creativity and aesthetic evaluation of AI-generated artworks: bridging problems and methods from psychology to AI

**DOI:** 10.3389/fpsyg.2025.1648480

**Published:** 2025-09-02

**Authors:** Ivana Bianchi, Erika Branchini, Tiberio Uricchio, Ramona Bongelli

**Affiliations:** ^1^Department of Humanities (Section Philosophy and Human Sciences), University of Macerata, Macerata, Italy; ^2^Department of Human Sciences, University of Verona, Verona, Italy; ^3^Department of Information Engineering, University of Pisa, Pisa, Italy; ^4^Department of Political Sciences, Communication and International Relations, University of Macerata, Macerata, Italy

**Keywords:** human creativity, AI creativity, human and AI-generated artworks, AI-generated art aversion, pleasure and interest model of aesthetic appreciation

## Abstract

This paper contributes to the debate on creativity, art, and artificial intelligence (AI) by integrating insights from cognitive psychology and empirical aesthetics into the field of AI, with the goal of inspiring novel empirical research. We focus on two main topics. First, we examine the indices used in psychology to operationalize *creativity* in closed-ended and open-ended tasks, with the aim not only of demonstrating the multidimensionality involved in defining creativity, but also of stimulating reflection on the benefits that might arise from developing a similar standard set of indices to test AI scoring models for assessing creativity (of both human and AI-generated responses). Second, we focus on the situation in which the creative products generated by AI are works of art, and on their aesthetic evaluation by non-expert human observers. Bridging the literature developed in psychology of art and empirical aesthetics with the literature on AI, a number of questions emerge, regarding the bias about the “expected style” of AI-generated art, and possible variables that play a role in aversion to AI-generated art. They all suggest possible future empirical research directions.

## 1 Introduction

There is considerable debate in current journals on creativity and artificial intelligence (AI)—the joint search of “creativity” and “AI” in Google scholar returned 18,200 scientific articles in the period 2020–2025 (data accessed on May 27, 2025). The discussion is multifaceted and touches on various issues (for an overview, see Elgammal, 2019; Cetinic and She, 2022; Du Sautoy, 2020; Liu et al., 2025; Manovich, 2022; Schröter, 2019; Yang and Xu, 2025). As Arielli and Manovich (2022) point out, many of these questions stem from an anthropocentric perspective on creativity that assumes that AI mimics human performance whereas, conversely, conceptualizing creativity in relation to AI within a non-human paradigm might illuminate the debate in new ways (see also Landers, 2025; Mazzone and Elgammal, 2019).

We see the advantage of exploring the topic from a non-anthropocentric perspective. At the same time, however, we believe that there are still ample opportunities to link research developed in psychology on human creativity to the ongoing debate on creativity and AI, in order to stimulate new perspectives from which to approach the topic and new empirical research. This paper contributes to these goals by focusing on two topics: first, the definition and operationalization of “creativity” in the psychology and AI literature; and second, the aesthetic appreciation of AI-generated art. In this context, aesthetic appreciation is defined as the response of liking (in terms of beauty and other possible scales, such as complexity, realism, effort, authenticity, etc.) in ordinary observers, as is commonly defined in the literature on the psychology of art. In other words, we are not dealing with evaluations made by artists, art critics, or other professionals of this sort. We are simply interested in comparing the reactions of naïve observers to human- and AI-generated artistic products, which is also the goal of all the literature cited in this paper. Understanding the cognitive biases behind the public’s common reaction to AI-generated artistic works is a timely topic that allows us to discover interesting aspects of the human mind and identify areas for improvement from the perspective of AI developers.

In reviewing the literature on these two topics, we adopt a specific point of view and do not address other topics, such as the definition of art and works of art. These are interesting subjects, but totally beyond the scope of this paper. Our overarching goal is to apply ideas and methods from cognitive psychology and empirical aesthetics to the current debate about AI creativity and AI-generated images that mimic artistic works. We also aim to identify new questions and future directions for empirical studies inspired by this perspective.

Before developing these two main topics in detail (see sections “Adopting indices used to assess human creativity in psychology and cognitive science to develop metrics/models for assessing AI-generated creativity” and “Empirical Aesthetics”) some preliminary considerations are necessary.

## 2 Some preliminary clarifications

### 2.1 Product versus process

First, it is crucial to clarify that the perspective adopted in this paper in no way implies that an AI capable of learning to produce highly regarded new paintings in the style of Mondrian (Noll, 1967), to generate Chinese landscapes so convincingly as to deceive observers (Xue, 2021), or to create a beautiful “next Rembrandt” (Sovhyra, 2021) follows the same *processes* as human artists. Even AICAN (Artificial Intelligence Creative adversarial Network), that is, an AI algorithm designed not simply to emulate an established style (as GAN algorithms tend to do), but to autonomously generate something new based on existing works (Elgammal, 2019; Elgammal et al., 2017), does not intend to reproduce the *process* that leads human artists to create their artworks.

Any human-generated artwork is the result not only of the human cognitive apparatus that supports its creation, but also of the personal and cultural (i.e., historical, conceptual, and symbolic) journey that led a particular artist to create a particular artwork. To put it in Yang and Xu (2025, p. 2) terms, human creativity is typically a highly personalized process, often emotion-driven and full of uncertainty and uniqueness (Garcia, 2024), with inspiration often being spontaneous and unpredictable. Consistent with this, it often has a self-expressing meaning and is not necessarily oriented towards external judgment (i.e., has a social meaning), while AI creativity relies or a computation-driven systematic process of analyses and reorganization of vast amounts of data (see also Runco, 2025). AI simply mimics the final *product* on a formal level (see also Arielli and Manovich, 2022; Hertzmann, 2020), corresponding to the minimization of a cost function of similarity to a known distribution of images (Goodfellow et al., 2020; Rombach et al., 2022) or, in the case of ICAN, generates a novel object that represents an “optimal” point between imitation and deviation from existing styles. AI has been successfully applied to design new paintings (Noll, 1967; Sovhyra, 2021; Xue, 2021), architecture with precise styles (e.g., Newton, 2019), to write poetry (e.g., Köbis and Mossink, 2021), novels (e.g., Green, 2020) and to complete the unfinished musical works of masters (e.g., Beethoven’s unfinished 10th Symphony was completed in “Beethoven X—The AI Project,” a collaboration between musicologists, composers, and computer scientists who fed the AI all of Beethoven’s existing works and sketches for the 10th Symphony; similarly, Schubert’s Unfinished No. 8 Symphony was completed by Huawei’s AI, which worked with Lucas Cantor to arrange these melodies into an orchestral score in Schubert’s style). In general, the creation of art using AI is nowadays a common practice for many video artists and sound artists, and even in film productions.

Since the focus in our paper is on the product, not on the process, questions that are interesting from a process point of view—for example, concerning consciousness and intentionality (Aru, 2025; Manu, 2024; Mikalonyté and Kneer, 2022; Moura, 2024; Redaelli, 2025)—are not relevant to the analyses addressed here.

### 2.2 The role of previously learnt knowledge

The second clarification concerns the role of previously learned knowledge in AI creativity. All the convincing products cited above come from the recombination of features, or configurations of features, learned by the AI during an initial training phase. This training phase, carried out on large databases, allows the AI to learn deeply, leading to new instances (i.e., not present in the original database) of the same types of objects used in the training.

The emphasis on the role of prior knowledge in AI-generated artefacts is often explicitly contrasted with the “novelty” that characterizes human creative responses and is used to deny the creative nature of the AI-generated products (e.g., Runco, 2023, 2025). However, framing the analysis in these terms overlooks the importance of prior knowledge in various theoretical models of creativity (e.g., Chaudhuri et al., 2024; Meyer and Pollard, 2006; Tromp and Glăveanu, 2023; van Welzen et al., 2024) and also in specific training procedures used to stimulate creativity (e.g., Birdi, 2016, 2020; Huo, 2020; Jones-Chick et al., 2022; Kienitz et al., 2014; Ritter et al., 2020; Sun et al., 2016). These procedures encourage individuals to make unusual associations between information “stored” in their knowledge bag. Unusual associations are typically thought of as remote associations, that is associations between elements that are “remote” in our usual representational network (for the importance of remote associations in the production of human creative responses, see Mednick, 1962; Kenett, 2019; Kenett and Faust, 2019; Olson et al., 2021; Toivainen et al., 2019; Wu C. L. et al., 2020). Encouraging the search for remote (rather than close and ordinary) associations is usually achieved by changing the default way in which individuals access their knowledge, for example, by asking them to focus on the properties of the parts of the object (or problem or situation) rather than on the properties of the object as a whole (Huo, 2020), or to turn the object’s properties in their opposites (e.g., Bianchi and Branchini, 2023; Branchini et al., 2021). However, in each case these strategies operate on the knowledge of the thinker, that is, on previously learnt material. Also atypical and unusual (because distant) connections presuppose linking concepts, features, and elements that were already present in one’s mental organization. In other words, just as AI relies on its training data, humans draw on their accumulated knowledge, experience (including training experience) and cultural heritage as input data when creating something new. By emphasizing the importance of prior knowledge in human creativity, we are not denying the existence of differences in the processes that lead to the recombination of this prior data. As we clarified in the previous section, we give for granted that these processes differ. We simply want to acknowledge the importance of prior knowledge as a fundamental ingredient in both human- and AI-generated creative products. Once this has been clarified, then a new question raises, that is, the question of how, if at all, the idea of transcending the accumulation of existing knowledge, matter, and concepts applies to products made by AI. This idea applies to human art, which transcends existing knowledge by offering unique perspectives, challenging established ideas, and prompting new ways of seeing the world, ourselves, and art itself. Can this apply to AI-generated art? How can we identify and measure this component of transcendence?

## 3 Adopt indices used to assess human creativity in psychology and cognitive science to develop metrics/models for assessing AI-generated creativity

Many classic definitions of creativity developed within cognitive science state that creativity is the ability to generate ideas that are both novel/original and effective, that is, appropriate and useful (e.g., Amabile and Tighe, 1993; Beghetto and Kaufman, 2022; Bruner, 1962; Kaufman and Sternberg, 2010; Mumford, 2003; Runco, 2012; Runco and Jaeger, 2012; Simon, 2001; Stein, 1953; Sternberg and Lubart, 1999). In line with this definition, psychologists have modelled the creative process as an interaction between a generative component—which supports the production of ideas—and an evaluative component—which concerns the selection of the ideas that seem most promising in terms of feasibility and potential success (e.g., Basadur et al., 1982; Ellamil et al., 2012; Finke et al., 1992; Hao et al., 2016; Rietzschel et al., 2019; Zamani et al., 2023).

Interest in the potential of AI to support the *generative phase* is certainly a major theme in the literature on AI, widely focused on developing and testing AI creative performance in comparison to human creative performance (e.g., Hubert et al., 2024; Ismayilzada et al., 2025; Koivisto and Grassini, 2023; Stevenson et al., 2023; Tian et al., 2023).

However, another interesting question is whether AI can also support the *evaluative phase*, or whether humans remain the gatekeepers, assessing the value and potential of AI creations. The latter position is suggested, for example, by Agrawal et al. (2017), Karimi et al. (2018), Magni et al. (2024), van Esch et al. (2019) and von Krogh (2018). At the same time, promising tests have been carried out in recent years on the possibility of using AI to assess the creativity of human-generated responses, in both verbal (e.g., Acar et al., 2021; Beaty and Johnson, 2021; Buczak et al., 2023; Dumas et al., 2021; Luchini et al., 2025; Patterson et al., 2024; Stevenson et al., 2020, 2023) and visual (e.g., Acar et al., 2025; Cropley and Marrone, 2025; Grassini and Koivisto, 2025; Patterson et al., 2024) creativity tasks. In some recent works the automated assessment was extended, comparatively, also to AI-generated responses (e.g., Chakrabarty et al., 2025; Hubert et al., 2024; Ismayilzada et al., 2025; Kern et al., 2024; Orwig et al., 2024; Stevenson et al., 2023). The goal in all cases is to develop automated scoring systems that mimic human classification of responses.^1^

In the next section, rather than comparing the performance of humans and AI on specific tasks, we will take a step back and consider the *types of tasks* and *indices* that are typically used in psychology to assess human convergent and divergent creativity (see Table 1). We will then discuss how this can inspire the development of new automated metrics and models to evaluate creativity in AI- and human-generated responses.

**Table 1:** Summary of the measures typically used in psychology to assess human creativity in relation to closed- and open-ended tasks.

Measure	Closed-ended tasks (CET)	Open-ended tasks (OET)
Success rate	✓	–
Fluency (Number of attempts made, in CET/of ideas produced, in OET)	✓	✓
Metacognitive feelings of certainty/uncertainty (feeling of confidence or rightness)	✓	✓
Feeling of being close to the solution (i.e., feeling-of-warmth) in CET/close to find a good idea in OET	✓	✓
Analyses of drawings produced during the search phase	✓	✓
Neurophysiological states accompanying or preceding the emergence of the solution in CET/accompanying the idea generation in OET	✓	✓
Eye-tracking movements	✓	✓
Flexibility (number of categorically different ideas)	–	✓
Originality: Quantitatively (number or proportion of unique responses) Qualitatively (expert ratings using the Consensual Assessment Technique [CAT])	–	✓
	–	✓
Usefulness or utility (real-world applicability of an idea): Feasibility, i.e., the practicality of an idea (expert ratings using the CAT) Value, i.e., the effectiveness of an idea in achieving a goal or reducing economic costs (expert ratings using the CAT)	–	✓
	–	✓

See the main text of the paper for a discussion of these two types of tasks and methods.

### 3.1 Transferring psychological constructs from convergent and divergent thinking tests to AI-generated responses

Classifying responses as creative or not (or as creative to varying degrees) has been a challenging topic for psychological research since it is not straightforward. The complexity of the classification becomes particularly evident when *open-ended tasks* are involved, that is, tasks that admit many alternative solutions and that relate to divergent thinking, while it is easier when creative convergent thinking and *closed-ended tasks* are concerned (for these definitions see Guilford, 1967a).

**Figure 1:**
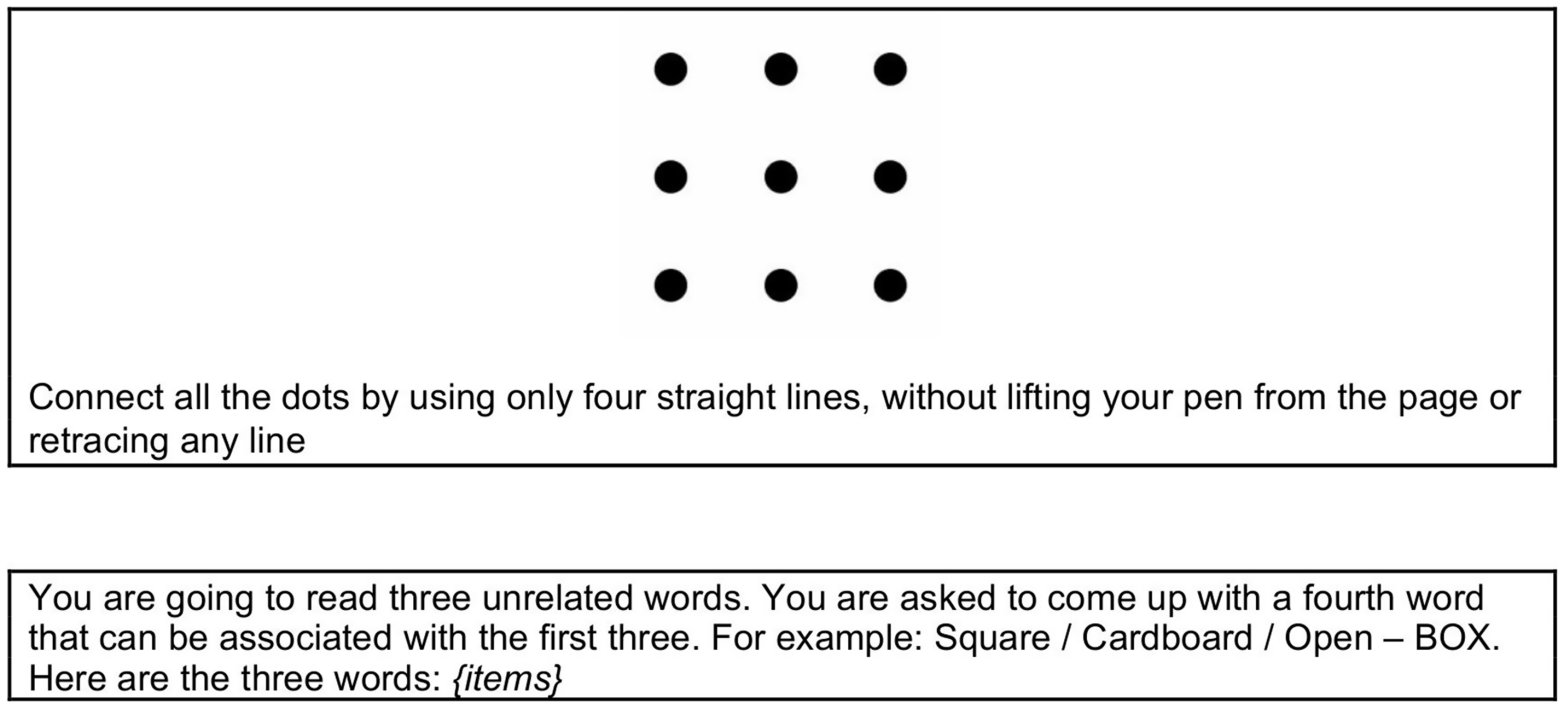
Two classic closed-ended tasks used in psychology: a visuo-spatial task (top box), i.e., the nine dots problem (Maier, 1930), and a verbal task (bottom box), i.e., the remote associates test (Mednick and Mednick, 1967).

Closed-ended tasks are characterized by having one correct solution which is usually not obvious and not easily accessed due to some unnecessary constraints introduced by the human mind in the initial representation of the situation/problem. Typical closed-ended tasks used in psychology are visual–spatial problem-solving tasks (e.g., Gilhooly and Murphy, 2005; Webb et al., 2021) or verbal problem-solving tasks (Webb et al., 2021) (Figure 1).

With closed tasks, there are simple and accessible ways to assess participants’ performance, the most straightforward being the success rate, that is, the ability of the participant to find the correct solution. These kinds of problems are structured to push the observer towards an initial representation that cannot lead to the correct solution. Finding the correct solution manifests that the problem solver has been able to overcome the initial fixation and restructure their representation of the problem in a novel and less default—in this sense more creative—representation (Danek, 2018; Danek et al., 2020; Graf et al., 2023). Various other measures of creativity can complement the success rate, such as the number of attempts made, for example. In convergent creativity and closed-ended tasks, the best performance is associated with reaching the solution as soon as possible, that is, the less attempts made, the more creative one is considered (e.g., Bianchi et al., 2020; Branchini et al., 2016; Öllinger et al., 2017). Other additional indexed aim to reveal the mental path followed by the participant while searching for the solution. For example, by analyzing the linguistic expressions used when thinking aloud in small groups, psychologists can track metacognitive aspects such as participants’ feelings of certainty or uncertainty, and how close they feel to finding the solution (Danek and Salvi, 2020; Danek and Wiley, 2017; Laukkonen et al., 2021; Salvi et al., 2016; Threadgold et al., 2018; Webb et al., 2016; Zedelius and Schooler, 2015). Other indexes concern the features of the drawings participants sketch while searching for a solution (e.g., Bianchi et al., 2020; Branchini et al., 2016; Fedor et al., 2015; Öllinger et al., 2017), or neurophysiological states that co-occur with or precede the emergence of the solution (e.g., Danek et al., 2015; Danek and Flanagin, 2019; Salvi and Bowden, 2024), and eye movements (e.g., Bilalić et al., 2021; Ellis et al., 2011; Ellis and Reingold, 2014; Knoblich et al., 2001; Xing et al., 2018, 2019). Collecting all this information requires technical mastery and can be time-consuming, but it is methodologically sound in terms of objectivity. All these measurements (except eye tracking and neurophysiologic responses) could be in theory applicable in the context of analyses of AI creative performance. For example, we could present puzzles to Large Language Models / Vision Language Models that they could solve using chain-of-thought and explanations, and in this way could track the evolution of their attempts till they reach the solution.

**Figure 2:**
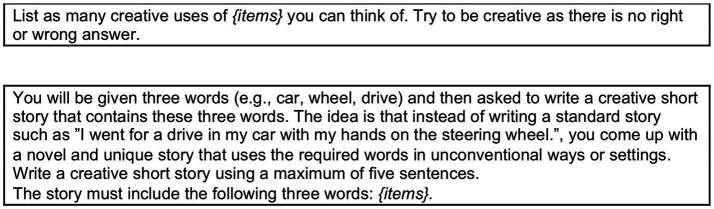
Two classic open-ended tasks used in psychology: the alternate uses task (Guilford, 1967b) (top box) and the five sentence creative story task (Prabhakaran et al., 2014) (bottom box).

When responses to an open task are concerned, the situation becomes more complicated also for the analyses of human creativity. Classic examples of open-ended tasks used in psychology studies are the Alternate Uses Task (AUT), or the Five Sentence Creative Story task (Figure 2), where 2–3 min time limits are usually set. The creation of an artwork is also an obvious example of open-ended task, since there are many alternative possible solutions.

Assessment of creativity measurement with this kind of tasks is more complicated, since there is no correct response and therefore success rate does not represent a meaningful measure of performance. The same measures used to capture participants’ mental paths with closed-ended tasks can be also used for open-ended tasks (e.g., analyses of drawings produced in the search phase, analyses of thinking aloud processes, metacognitive feelings—see Ball and Christensen, 2009, 2020; Ball et al., 2021; Christensen and Friis-Olivarius, 2020), but with regard to the classification of the product, the following measures are usually considered: *fluency*, *flexibility*, *originality* and *utility* (see Beaty and Johnson, 2021; Bellaiche et al., 2023a; Brosowsky et al., 2024; Forthmann et al., 2020; Long and Pang, 2015; Primi, 2014; Primi et al., 2019; Reiter-Palmon et al., 2019; Silvia et al., 2008; Snyder et al., 2019; Weiss and Wilhelm, 2022).

*Fluency* refers to the number of ideas produced by the participant. Although fluency alone does not define creativity (e.g., Rietzschel et al., 2006), the assumption is that the more ideas produced, the higher the possibility that a creative idea emerges, especially because the first ideas are the less creative and the more the person persists in ideation, the more they arrive at more creative ideas (Lucas and Nordgren, 2020).

*Flexibility* refers to the number of categorically different ideas. The assumption is that the more different categories involved, the more creative the person is. For instance, in the Creativity Assessment Packet (Williams, 1993), where a series of drawings must be produced starting from fragments of lines or shapes, flexibility is measured in terms of different categories of objects drawn. If a person draws a living being in one drawing, a landscape in another, a symbol in yet another, and a useful object in another, this is considered an indication of flexibility. A person whose drawings fall within the same category type (e.g., landscapes) demonstrates less flexibility. Likewise, in AUT, response patterns classified as more flexible are those that traverse various conceptual spaces—e.g., using a “brick” as a paperweight, step to gain height, and sidewalk chalk, rather than imagining uses which variously concern holding things in place (e.g., a paperweight, bookend, doorstop) (Hass, 2017; Nijstad et al., 2010).

*Originality* refers to the degree of uniqueness of the idea produced, and the evaluation of this aspect is more challenging. Originality can be described quantitatively, in terms of the numbers of unique responses (initially formalized by Wallach and Kogan, 1965). The assumption is that the smaller the proportion of participants who come up with the same idea, the more original the idea is (e.g., DeYoung et al., 2008; Hocevar, 1979; Putman and Paulus, 2009; Webb et al., 2021—for a critical view of uniqueness scoring and ways to address its problems see Silvia et al., 2008). However, originality is traditionally measured qualitatively using Amabile’s (1983, 1996) Consensual Assessment Technique (CAT). In this technique, domain experts independently rate responses on a a scale ranging from “not at all creative” to “very creative” (Silvia et al., 2008; see also Baer and Kaufman, 2019; Kaufman et al., 2008).

*Usefulness*, or *utility*, corresponds to the real-world applicability of an idea and is defined in terms of both *feasibility*—that is, the practicality of an idea (Poetz and Schreier, 2012; Rietzschel et al., 2010, 2019)—and *value* (Litchfield et al., 2015)—which lacks a universal definition but is mostly related to the *effectiveness* of an *idea in achieving a goal* or in terms of *economic costs* (e.g., Ford and Gioia, 2000). Usefulness is also usually measured with the CAT. However, because usefulness is difficult to score, raters are generally only asked to score originality, despite usefulness playing an important and distinct role in evaluating creativity (Diedrich et al., 2015; Rietzschel et al., 2019).

The problem with CAT scoring is, first, its reliability. While some studies have proven its validity (Amabile, 1982; Kaufman et al., 2007; Long and Wang, 2022; Myszkowski and Storme, 2019), others have highlighted the impact of personal or cultural biases on this subjective classification (e.g., Blair and Mumford, 2007; Dailey and Mumford, 2006; Ivancovsky et al., 2019; Toh and Miller, 2016).^2^ Additionally, the consensus reached depends on the raters involved (e.g., Cseh and Jeffries, 2019; Forthmann et al., 2017; Plucker et al., 2019; Reiter-Palmon et al., 2019; Zhou et al., 2017). Second, the CAT technique certainly requires time and efforts on the part of the raters, and costs for the researches. For this reason, as mentioned at the beginning of this section, automated scoring methods have recently been developed that have the potential to assist researchers and/or educators in assessing human responses. The classic procedure is to train the AI using a sample of human-generated responses scored by independent raters, and then to test different machine learning models for automated scoring.

Many of these studies have shown that AI scoring is as accurate and reliable as human raters (e.g., Beaty and Johnson, 2021; Cropley and Marrone, 2025; Dumas et al., 2021; Kern et al., 2024; Hubert et al., 2024; Luchini et al., 2025; Sun et al., 2024). However, when we compare these studies, a question emerges: What criteria are used to measure creativity? Stevenson et al. (2023) compared human and open AI performance on the GPT-3 and GPT-4 in the Alternate Use Test (AUT), evaluating responses in terms of *originality*, *usefulness*, *surprise*, and *flexibility*. The same task was used by Hubert et al. (2024) in addition to two other open-ended tasks (the Consequences Task and the Divergent Association Task) to compare human and GPT-4 performance in terms of *fluency*, *originality* and *elaboration*. Ismayilzada et al. (2025) used the Five Sentence Creative Story task to compare the performance of humans and of 60 different Large Language Models in terms of *novelty*, *surprise*, *value*, *lexical* and *semantic diversity*. The Five Sentence Creative Story task was also used by Orwig et al. (2024) to compare the performance of humans and GPT-3 and GPT-4, but the responses were scored in terms of a general *creativity* rating. Guzik et al. (2023) compared ChatGPT (GPT-4) and human creative performance at the Torrance Tests of Creative Thinking by analyzing *fluency*, *flexibility* and *originality*.

A standardized system of creativity indices (dependent variables) would allow researchers to more easily compare the results of their studies and effectively compare the generative performance of different AI models with respect to different tasks. Furthermore, since some cited papers use AI to generate and score responses (Hubert et al., 2024; Ismayilzada et al., 2025; Kern et al., 2024; Orwig et al., 2024), reaching a consensus on the definitions used to operationalize fluency, flexibility, originality/novelty, feasibility, and value would help identify the parameters used in these automated AI scoring systems. We are not suggesting that this is an easy goal to achieve—on the contrary—only that it could be valuable. The brief overview provided in this section will hopefully also help to highlight the risks of using a generic “provide a rating of creativity” prompt (e.g., Orwig et al., 2024; Seli et al., 2025), since the definition of creativity is multifaceted and encompasses many dimensions—as reflected in the struggle of cognitive scientists to find appropriate ways to measure it. Using precise prompts when defining the scoring criterion seems preferable, both for human raters and AI models. Several studies have gone in this direction (e.g., Hubert et al., 2024; Ismayilzada et al., 2025; Kern et al., 2024; Stevenson et al., 2023).

## 4 From empirical aesthetics to AI

Let us now specifically focus on the situation in which the “creative” products generated using AI are artworks and on their aesthetic evaluation by human observers. Relatively little is known about (a) whether people can accurately attribute what they observe to the category of human-generated art versus AI-generated art (e.g., Chamberlain et al., 2018; Gangadharbatla, 2022; Hong and Curran, 2019; Samo and Highhouse, 2023; Velásquez-Salamanca et al., 2025) and (b) how people aesthetically evaluate AI-generated artworks (e.g., Agudo et al., 2022; Bellaiche et al., 2023b; Chiarella et al., 2022; Gangadharbatla, 2022; Hong and Curran, 2019; Horton et al., 2023; Messingschlager and Appel, 2025; Neef et al., 2025; Ragot et al., 2020; Velásquez-Salamanca et al., 2025; Wu Y. et al., 2020). However, it is not difficult to see how these are becoming hot topics for research. In a recent empirical study, Yang and Xu (2025) explored the core dimensions of AI creativity *from the audience’s viewpoint*, uncovering audience biases against AI creativity. Namely the more AI was said to be involved in a creative process, in collaboration with a human being, the less the results were perceived as deep, authentic and attractive. However, it was perceived as more original. Here, we will add to these biases about AI creativity, other “biases” that emerge from a broad review of existing literature. For each of them, we will highlight open questions that may inspire interesting directions for future research aiming to discover the cognitive sources of these biases.

### 4.1 The “typical style” of visual artworks attributed to AI

In various studies in which participants were exposed to images of both abstract and figurative paintings purportedly generated by either AI or humans, it was found that figurative images were more often attributed to humans, whereas abstract images were attributed to AI (e.g., Gangadharbatla, 2022, study 1). Alternatively, some findings suggest that figurative art was attributed indifferently to AI and humans, while abstract art was preferentially attributed to AI (see Chamberlain et al., 2018, study 1).

Empirical aesthetic research has shown that non-art experts tend to have a clear preference for figurative art over modern art, which is more often appreciated by individuals with greater familiarity with and expertise in art (Bimler et al., 2019; Knapp and Wulff, 1963; Leder et al., 2012; Leder and Nadal, 2014; Mastandrea et al., 2011, 2021; Pihko et al., 2011). The bias toward attributing abstract art to AI suggests an implicit appreciation of human art (since figurative art is generally preferred) and a devaluation of AI art (since abstract art is less preferred). This is further supported by studies in which aesthetic judgments of AI-generated compared to human-generated art were explicitly solicited, as discussed in the following section. However, the aspect we want to highlight here is that these attributions suggest *that people implicitly assume that abstract art is the typical “style” of AI-generated images*. This conclusion is consistent with some findings emerged from Chamberlain et al. (2018), where participants were first asked to attribute images of artworks to either humans or AI and later to explain “How did you decide if a work was computer-generated?” (free-response task). Thematic analyses of participants’ responses revealed that there were relatively few references to content or intentionality; instead, responses focused primarily on surface and structural aspects of the artworks. Namely, the most common justifications for categorizing images as computer-generated were bright or artificial colors and rigid, straight, regular shapes and lines. Conversely, human-generated images were classified as such based on their appearance of being handmade, as evidenced by irregular, imperfect lines and a larger distribution of orientations, as well as the presence of brushstrokes (for this last aspect, see also Fuchs et al., 2015). Similarly, Noll (1967) and Schröter (2019) found that ordered and regular distributions of elements were associated with AI-generated art.

Gangadharbatla (2022, p. 15) suggests that this typical “style” of AI (associated more with simple and regular composition) may have originated from early experiences of AI generation, with algorithms that were not as sophisticated as current ones, leading people to expect that AI could only produce simpler (i.e., abstract) artworks, not compelling figurative artworks. However, following projects such as the next Rembrandt (Sovhyra, 2021) and the development of databases of high-resolution human faces and landscapes produced by the latest generation of AI algorithms (Rombach et al., 2022), this limitation has been overcome. It seems promising to investigate whether participants exposed to these updated types of AI-generated images—either because of their professional or educational background, or because they are exposed to images such as the next Rembrandt during a training phase—still exhibit the *abstract = AI versus figurative = human* equation, or whether this association would be significantly reduced. Such data would help shed light on the roots of the prototypical idea of AI-generated art that people seem to hold. If it is simply due to a mere exposure effect, this should be a very transient bias that disappears after exposure to a training session that allows participants to acknowledge the possibility of AI generating realistic figurative images. If the bias persists, it would suggest that the bias is related to other factors (perhaps linked to a broader technology vs. anthropological cognitive dimension) that would be interesting to understand.

### 4.2 The diminished value of AI-generated art when compared to human-generated art

Psychology studies on the aesthetic evaluation of human-generated artworks have shown that people’s evaluation can be modified by providing contextual information, such as historical information about the artist (e.g., Fischinger et al., 2020), the style of the artwork (e.g., Russell, 2003), the intentional message the artist wanted to convey (e.g., Millis, 2001), the thought process involved in the innovation displayed by the artwork (Bianchi et al., 2025), and the skills required to create the artwork (e.g., Hodge, 2012). This information increases non-art experts’ understanding of and interest in the artwork, more than the pleasantness of the artwork (e.g., Belke et al., 2006; Bianchi et al., 2025; Jucker et al., 2014, experiments 2a, 2b; Leder et al., 2006, experiment 1).

Research has only just begun to connect the fields of empirical aesthetics and AI, but the role of contextual factors also seems central for research on AI-generated art. Studies comparing evaluations of human-generated and AI-generated art have found that providing contextual information about the creator (human or AI) changes participants’ appreciation of the artwork. This effect is typically assessed using experimental designs in which the same images are presented in two different conditions: one in which participants are unaware of the authorship and another in which they are informed that the artwork was created by a human or by “technical” tools; or by presenting the same images in one condition labelled as AI-created, and in another condition as human-created. These studies show, for example, that viewers rated images labelled as having been created in Photoshop as less aesthetically pleasing than the same images labelled as having been taken from an art gallery (Kirk et al., 2009). Similarly, participants rated the same images of painting as more beautiful when they were attributed to human creators than when they were said to have been generated by robots (Chamberlain et al., 2018; Di Dio et al., 2023) or AI machine learning algorithms (e.g., Hong and Curran, 2019; Horton et al., 2023; Millet et al., 2023; Ragot et al., 2020; Wu Y. et al., 2020). The bias against AI-generated art persists even when it is emphasized that the artwork was created in a collaborative production between humans and AI (Horton et al., 2023, study 6; Messer, 2024, Yang and Xu, 2025) and is not exclusive to the visual arts. Participants rate the quality of a music lower when they were informed it was AI-generated rather than human-made (e.g., Agudo et al., 2022; Hong et al., 2020, 2022; Moffat and Kelly, 2006; Shank et al., 2023). Similar results were observed in dance choreography (Darda and Cross, 2023). Neef et al. (2025) suggest that lower appreciation of AI-generated art emerges in particular when it is compared to human-generated images and not independently of this comparative framework (this is also highlighted in Horton et al., 2023).

In the following subsections we will focus on visual art, we outline five possible factors underlying this aversion that have been identified in various literature (leaving aside the role of individual differences) and that prefigure possible promising lines of future research to be developed.

#### 4.2.1 Is the aversion mitigated by the artistic style of the painting considered?

Aversion to AI-generated art may stem from a broader phenomenon known as “algorithm aversion” in decision making (Castelo et al., 2019; Dietvorst et al., 2015; Shaffer et al., 2013; Yeomans et al., 2019). However, the literature on decision making also suggests that in certain cases where decisions require objectivity, lack of bias, and neutrality, an opposite phenomenon occurs, referred to as “algorithmic appreciation” (Sundar, 2008). This phenomenon consists of an overestimation of machine performance compared to human performance (see Castelo et al., 2019; Liu and Wei, 2019; Logg et al., 2019). The algorithmic appreciation phenomenon suggests that certain characteristics stereotypically attributed to machine performance can, under certain circumstances, elicit positive attitudes in observers. It has already been found that the use of AI led to more positive evaluations of intangible products, such as songs, compared to tangible products, such as paintings (Tigre Moura et al., 2023), but whether this might be related or not to algorithmic appreciation has not been discussed in the original paper. One relevant question is whether the appreciation of tangible, AI-generated art (i.e., paintings) might also benefit from algorithmic appreciation. For example, AI-generated paintings might be evaluated more positively in genres that require precision, accuracy, and programmed sequences of elements, and less positively in others.

Some studies have recently appeared on the different abilities of GenAI to imitate different artistic styles (Asperti et al., 2025; Ha et al., 2024; Tang et al., 2025). For example, Asperti et al. (2025) found that AI generative models appear to be more adept at imitating artistic styles such as Impressionism, Cubism, Dadaism, and Futurism, which emphasize “abstraction, bold forms, and expressive brushwork” (p. 14), whereas they face greater challenges when attempting to imitate Renaissance, Baroque, Rococo, and Naive art styles. The inability to distinguish between human-generated and AI-generated art for some styles (reflecting gen-AI’s success at imitation) is not the same as appreciation. As we have seen in the previous pages, participants often depreciate the same artworks when they know they are AI-generated, even though they cannot distinguish them from human-made artworks. It would be interesting to study whether aversion to AI-generated art is weaker for certain styles. This aversion may be weaker for styles, such as Op Art, Cubism, and Abstract Expressionism versus Impressionism, Renaissance and Baroque Art. This hypothesis is based on the implicit assumptions participants have about the “typical style” of visual art attributed to AI, as discussed in a previous section (section 4.1). A comparative analysis of the appreciation of AI-generated art in different styles would allow us to discover various implicit assumptions.

#### 4.2.2 Is the aversion mitigated by witnessing the production process?

Based on the few studies that have examined the impact of personal beliefs on the appreciation of AI-generated art, we know that the bias against AI-generated art is primarily driven by participants who do not attribute creative skills to AI (e.g., Agudo et al., 2022; Chamberlain et al., 2018, study 2; Di Dio et al., 2023; Hong et al., 2020), have a negative attitude towards AI (Neef et al., 2025), and hold anthropocentric beliefs about creativity, that is, they believe creativity is a uniquely human trait—which leads them to see less creative value in, feel less awe for, and be less likely to purchase AI-generated art (Millet et al., 2023). The negative bias towards AI-generated artworks was also found to depend on personality traits (e.g., Grassini and Koivisto, 2024) and cultural differences (e.g., Wu Y. et al., 2020). All these findings highlight the importance of more frequently assessing individual factors in future studies that aim to test the appreciation of AI-generated art.

With this premise in mind, an interesting hypothesis that merits further consideration is that exposure to the production process may improve evaluations of AI-generated art. Witnessing the art production process may enhance participants’ appreciation of the “ability” required by AI to create a painting, sculpture, or a piece of music, paralleling the appreciation we have for human artists’ technical skills. This hypothesis is provisionally supported by the results of few, but interesting, studies (e.g., Chamberlain et al., 2018, study 2; Tresset and Leymarie, 2013). In particular, Chamberlain et al. (2018, study 2) demonstrated that the aesthetic response and artistic value of portraits created by robotic artists increased when participants could observe them at work. In one condition, participants were present while the robots drew portraits of individuals sitting in a chair (for full details on the robot used, see Tresset and Leymarie, 2013) and could interact with them by having their portrait drawn. In another condition, they were presented with the drawings and informed that they had been created by a robot. In a third condition, they received no information about how the drawings were created. The aesthetic ratings of the drawings were higher when participants assisted the robots while drawing as compared to both the condition where only information about the artist was provided without direct observation or interaction, and the condition where no information was given (leading participants to assume the portraits were made by a human artist).

A connected factor to consider is the anthropomorphisms that is brought into play by the process. For instance, activation of the motor and premotor cortices was found when participants were exposed to Lucio Fontana’s Cuts (Umiltà et al., 2012) or Franz Kline’s artworks—characterized by wide, marked traces of brushstroke (Sbriscia-Fioretti et al., 2013). These findings have been explained in terms of embodied simulation of the artist’s gestures during the perception of the artworks (see also Freedberg and Gallese, 2007; Oberman et al., 2007). In the context of AI, these findings raise the question of whether witnessing the art production process by AI might lead to increased engagement and a more positive evaluation of the generated output, and whether this is moderated by the amount of anthropomorphism prompted in the generative process (Waytz et al., 2014). The robots in Chamberlain et al. (2018) lacked humanoid visual characteristics, but the dynamics of their actions suggested them—particularly the robot’s alternating “looking behaviour” toward the person whose portrait was to be made and the drawing in progress. Direct exposure to the production action may activate the same mechanisms of motor simulation that occur when observing human actions.

#### 4.2.3 Is the aversion mitigated by information about the effort and time needed to create the artwork?

We know from psychology studies, that the aesthetic appreciation of human artworks also depends on the information provided to observers about the time the artist spent in direct contact with the artwork. The longer the contact, the higher the perceived value of the object (Newman and Bloom, 2012). Similarly, the more time and effort attributed to the artist in creating the artwork, the higher observers’ ratings of liking, quality, and value of the artwork (Jucker et al., 2014, study 3; Kruger et al., 2004).^3^ The effect of time has been explained through effort heuristics. Assessing quality can often be challenging—for instance, determining the monetary value of a painting or the scientific contribution of a paper or book. When this is the case, people use effort as a heuristic for quality. Effort is generally a reliable indicator of quality; all else being equal, paintings that have received prolonged attention from the artist, as well as papers/books that have required extensive time to create and revise, usually result in better work. This logic supports the existence of the heuristic. However, like all heuristics, it can sometimes lead to errors. Moments of inspiration, for example, can occasionally result in unexpectedly quick and optimal outcomes.

How can we relate this to AI-generated art? If participants assume by default that AI is “quick” in doing what it does—and robots and AI systems are typically perceived to reduce effort and labor for humans (see Bechwati and Xia, 2003; Kruger et al., 2004)—this may contribute to the negative aesthetic bias toward AI-generated art. Some empirical findings support this hypothesis. For example, Horton et al. (2023) found that participants evaluated AI-labeled artworks as having taken less time to produce and as being less creative and worth less money. Magni et al. (2024, Studies 2–3) found that participants rated AI as exerting less effort than humans when performing creative tasks (e.g., designing marketing campaign posters and generating business ideas). These attributions directly correlated with negative evaluations of creativity.

Would judgments change if observers were informed that, conversely, the creation of the artwork required significant time to AI or involved the interlocking action of multiple AI networks? These are pertinent questions for empirical research. To the best of our knowledge, there is currently very little literature on this topic. Magni et al. (2024, study 4) found that in a condition where both information about the creator (AI versus human) and effort (low versus high) were manipulated, participants associated the highest creativity ratings with the human-high effort condition, followed by the human-low effort condition, the AI-high effort condition, and, finally, the AI-low effort condition. These findings indicate that effort could not substitute for the effect of the creator’s identity. However, they also demonstrate an extension of effort heuristics to the domain of AI creativity. Indeed, time and effort were not irrelevant variables for judging AI products. Bellaiche et al. (2023b) found that the artworks that participants judged to have required high effort, received higher ratings of liking and beauty when they were attributed to humans. Conversely, the artworks that participants thought required low to moderate effort received higher liking and beauty ratings when attributed to AI.

#### 4.2.4 Is the aversion mitigated by emotional engagement with AI-generated art?

In discussing whether computers can replace human artists, Hertzmann (2018) argues that “art requires human intention, inspiration, and a desire to express something.” Various authors have emphasized that the aesthetic value of an artwork also lies in its capacity to evoke experiences charged with emotions that the creator felt and transferred into the artwork (e.g., Di Dio and Gallese, 2021; Pelowski et al., 2020, 2023). Framing the question in these terms seems to rule out any possibility of AI producing art since it is impossible for AI to transfer an experience or emotion “felt” by AI into the produced object (we can call this the *impossibility of being emotionally engaged by AI art* argument). In other words, when an artwork is created by a non-human entity, such as a computer, no human emotions can be expressed (e.g., Lu, 2005). Is this the reason why paintings labeled as AI-generated are rated by participants as less emotional than those attributed to humans (Demmer et al., 2023; Horton et al., 2023, study 1), and less awe-inspiring (Millet et al., 2023)? Or why participants exposed to identical pieces of video art or music report greater emotional arousal (“To what degree would you say that it aroused your emotion?”) when they believe the artist is human than when they believe it is AI (Agudo et al., 2022)? Or why articles written by an algorithm are considered more objective but also less emotionally engaging than those written by humans (Liu and Wei, 2019)?

On the other side, there is evidence that people can be emotionally engaged by AI art (*possibility of being emotionally engaged by AI art* argument). Bellaiche et al. (2023b) found that, for both images labeled as to human-created artworks and images labeled as to AI-created artworks, ratings of liking and beauty increased as the emotion rating (“To what extent does this artwork elicit an emotional response in you?”) increased. Furthermore, as Arielli and Manovich (2022, p. 16) point out: “The success of virtual pop stars in East Asian cultures (such as Hatsune Miku and several K-pop ‘avatar’ bands, some of them AI-driven) reveals how the public can emotionally engage with a fictional performer, follow them on social media, attend their concerts, and purchase merchandise depicting them. We could go as far as to say: fans do not love them despite, but actually because they are openly fake.”

Finally, we cannot help but notice that it is also a common experience that we sometimes appreciate a decorative pattern or the design of an object—such as a piece of furniture, a car, or a pair of shoes—without any emotional engagement with the feelings, experience, and intentions of the creator (*the indifference to emotional engagement* argument). These works can be pleasant, engaging, and entertaining in their own right.

We need further empirical evidence to clarify both the conditions that support an emotional response to AI-generated art and the necessity of an emotional engagement.

#### 4.2.5 Is the aversion better understood by analysing “liking” separately in terms of pleasure and interest?

Over the past decade, several models of aesthetic liking developed in the psychology of art and empirical aesthetics have conceptualized the idea of “liking” at two different levels (e.g., Graf and Landwehr, 2015, 2017; Leder et al., 2004, 2012; Leder and Nadal, 2014; Pelowski et al., 2017), by applying dual-process theories developed in social psychology and the psychology of reasoning (e.g., Chaiken and Trope, 1999; Gawronski and Creighton, 2013; Evans, 2006, 2008; Evans and Stanovich, 2013) to the analyses of aesthetic appreciation. Here, we refer to the Pleasure and Interest model of Aesthetic liking (PIA), developed by Graf and Landwehr (2015, 2017) for human-made art, as a framework for thinking about aesthetic appreciation in AI-generated art.

According to this model, aesthetic appreciation emerges from two hierarchical, fluency-based processes. The first level is based on an automatic, default process (“gut- response”) that results in an immediate affective response of *pleasure* or *displeasure* (Reber et al., 2004, p. 365; Strack and Deutsch, 2004; Winkielman and Cacioppo, 2001; Zajonc, 1980). The second level is activated when the observer engages in further controlled and effortful processing of the object, which involves an active and reflective interaction with the stimulus (Augustin and Leder, 2006; Belke et al., 2015; Pelowski et al., 2016; Reber, 2022). The decision to process a stimulus at this second level is determined by the interplay between the observer’s motivation and the pleasure or displeasure experienced at the first level. If observers experience displeasure at the gut level (“I do not like it: why is this art?”)—or disfluency, using the model’s terminology—they may be motivated to activate the second stage in order to gain a deeper understanding of the object. This second level of processing can lead to an experience of liking that is different from the first type (pleasure) and is referred to as “interest.” Interest arises when the viewer feels that the information they have discovered or learned has improved their fluency in processing the object. Not only do they feel that they have learned something about what they are observing (the artwork), but they also experience a change in their way of thinking. They perceive themselves as adopting a different, more analytical style of processing, paying attention to non-salient attributes, and feel an increased sense of mastery as a result of this transformation. Metacognitive aspects are embedded into this second level of liking (Alter et al., 2007; Bullot and Reber, 2013; Christensen et al., 2023).

Keeping this framework in mind when analyzing human response to AI-generated art may help to identify new questions and consider new methods for inquiring whether participants like or dislike AI art. To the best of our knowledge, the PIA model has only been considered by Bellaiche et al. (2023a). Namely, it inspired them to investigate participants’ responses to art labeled as AI-generated (compared to art labeled as human-generated) at a multidimensional level, asking participants not only for ratings about liking and beauty (which capture the first level of liking, pleasure), but also for rating of profundity (“How profound is the artwork?”), meaning (“To what extent do you find this artwork personally meaningful?”), story (“To what extent can you imagine a story being communicated through this artwork?), in addition to questions about the amount of time and effort they believed was involved in creating the artwork and the emotional response it elicited.

We believe that applying the PIA model directly to AI-generated art could inspire at least two new research directions. One direction would examine the surface-level features of AI-generated artworks that are appreciated. Do these features correspond to those appreciated in human-generated art, such as symmetry (Reber, 2002; Wurtz et al., 2008), contrast or clarity (Bornstein and D’Agostino, 1994; Halberstadt, 2006; Messinger, 1998; Reber et al., 1998; Reber et al., 2004; Song et al., 2021; van Geert and Wagemans, 2020; Winkielman et al., 2006)? Another research direction is to investigate whether exposure to information about the artist (i.e., AI), the concept behind the artwork (often encapsulated by its title), and the process leading to its creation would influence participants’ interest in the artwork, if not their perceived pleasure. Connected to this is exploring whether increased interest would also correspond to experiencing an improved sense of mastery and fluency in dealing with AI-generated art, that is, the metacognitive aspects involved in appreciation at the second level of the PIA model.

Table 2 summarizes the main biases toward AI creativity and the key research questions discussed in the previous sections.

**Table 2:** Key research questions related to some “AI creativity biases”.

Biases	Research questions
Typical style: participants implicitly assume that the typical style of AI-generated images is abstract art, i.e., simple and regular compositions, bright or artificial colors, and rigid, straight, and regular shapes and lines.	Is this bias rooted in prevalent exposure to early AI-generated images, and could it be corrected with extensive exposure to images resulting from the latest generation of AI algorithms? Does this bias reflect a broader underlying technological (precise, programmed, rigid) versus anthropological (imprecise, fantastical, flexible) divide?
Diminished value (Aversion to AI-generated art): participants tend to rate artistic performances (e.g., painting, music, and choreography) as less beautiful or aesthetically pleasing when attributed to AI rather than human creators.	Is this aversion mitigated by the type of art considered? For instance, AI-generated art may be viewed more positively in genres requiring precision, accuracy, and programmed sequences of elements. Is this aversion mitigated by witnessing the production process? Seeing how AI creates a painting, sculpture, or piece of music may enhance participants’ appreciation of the technical skills required (likewise, as with human artists). This effect may be further moderated by the amount of anthropomorphism prompted in the generative process. Is this aversion mitigated by information about the effort and time needed to create the artwork (the more time and effort, the more appreciation—as for human-generated art)? Is the aversion mitigated by emotional engagement with AI-generated art or is emotional engagement unnecessary? Can this aversion be better understood by analyzing “liking” in terms of pleasure and interest separately (as foreseen by dual models of aesthetic liking)? Does AI-generated art spark more interest than pleasure? Are the same stimulus-based features that are liked in human-generated art, such as symmetry, contrast, and clarity, also appreciated in AI-generated art? Does exposure to information about the artist, the artwork, or the process of its creation enhance interest? Does experiencing increased interest also imply that you feel more mastery and fluency in dealing with AI-generated art?

## 5 Discussion

The purpose of this paper was to contribute to the ongoing discussion of how we can operationalize the constructs of creativity (which was developed to define one of the most distinctive capacities of the human mind) and aesthetic appreciation when applied to AI creations. As clarified in the Introduction, we adopted an operational perspective bridging concepts and methods taken from the literature about creativity and art appreciation as developed in cognitive psychology and empirical aesthetics into the ongoing debate on creativity and AI, with the aim to stimulate new perspectives and questions, and suggest new directions for future empirical research.

We started with two clarifications. First, by reminding that making comparisons between human-generated and AI-generated products (concerning both creative responses in general, or artworks) does not imply that the processes leading to them are the same. Second, by pointing out that previously learned knowledge is fundamental to both human and AI creativity—therefore, we should not jump too quickly to the conclusion that AI responses cannot be novel simply because they are based on prior knowledge.

We then moved on to the first of the two main topics of the paper. We examined the indices used in psychology to operationalize creativity in closed-ended tasks, where convergent creative thinking is involved, and in open-ended tasks, where divergent creative thinking is required (section entitled “Adopt indices used to assess human creativity in psychology and cognitive science to develop metrics/models for assessing AI-generated creativity”). The goals of this section were (a) to show the multidimensionality involved in the definition of creativity, (b) to provide a systematic list of indices and a clarification of the corresponding aspects they are intended to capture, and (c) to stimulate reflection on the benefits of developing a standard set of indices (and of using similar operational definitions) for research testing the performance of automated AI scoring models to evaluate the creativity of both human-generated and AI-generated responses.

The second major theme of the paper (developed in the section entitled “From empirical Aesthetics to AI”) focused on the situation in which the creative products generated by AI are works of art, and on their aesthetic evaluation by human observers. Bridging the literature developed in psychology of art and empirical aesthetics with the literature and interest in AI, a number of new questions emerged. A first aspect focused on concerns the bias of associating abstract artworks with AI-generated art (and figurative artworks mainly with human-generated art, or both) and possible ways to empirically verify the factors underlying this bias. A second aspect concerns the diminished value usually attributed by participants to AI-generated art, compared to human-generated art. In reviewing the literature on the topic, we kept in mind some variables that have been shown in psychology to be effective in explaining participants’ aesthetic appreciation of human-generated art, and suggested a set of five questions that can be applied to the study of human appreciation of AI-generated art. These questions suggest possible future empirical research directions: Is AI-generated art aversion mitigated by the type of art considered? Is the aversion mitigated by witnessing the production process? Is it mitigated by information about the effort and time required to create the artwork? Is the aversion mitigated by emotional engagement with AI-generated art? Is it better understood by analysing “liking” separately in terms of pleasure and interest? To all these questions we hope future experimental research will find valid and intriguing answers.

## Footnotes

^1^For example, Cropley and Marrone (2025) used a Convolutional Neural Network (deep learning) to automate the scoring of figural creativity. They focused on the Creative Thinking-Drawing Production test (TCT-DP; Urban and Jellen, 1996; Urban, 2005), in which participants are presented with squared frames containing incomplete and irregular figural fragments (e.g., a dot, a dashed segment, a circle) and are asked to complete the drawings. Based on the TCT-DP manual, 14 criteria must be used to score responses, such as connection by theme (do participants integrate the individual elements into a thematic whole?), the use of perspective, the presence of boundary-breaking (do participants draw elements that extend beyond the squared frame?), or unconventional use of the figural fragments/elements. Cropley and Marrone (2025) trained AI using a sample of responses produced by participants and scored by two independent raters (with inter-rater agreement always >0.90). They tested various AI models of scoring, which varied from two levels of creativity (low and high) to seven levels of creativity (corresponding to the seven classes defined in the TCT-DP test manual). Despite the small dataset of images used by AI in the training phase (*N* = 414), very good accuracy scores for the automated scoring emerged, along with high levels of inter-rater agreement with human raters.

^2^Familiarity with the topic, for instance, can lead to overestimating the positive benefits and outcomes of an idea while underestimating the resources needed to implement it (e.g., Dailey and Mumford, 2006). Experts demonstrate higher interrater agreement in their evaluations than novices (e.g., Kaufman et al., 2008), but they also have a better understanding of the benefits of continuous (incremental) innovative ideas and more resistance toward discontinuous (radical) innovations, which are often better appreciated by non-experts (Moreau et al., 2001). The evaluative outcome also depends on personality factors. For instance, individual risk aversion and ambiguity aversion (Blair and Mumford, 2007; Toh and Miller, 2016) are associated with the selection of less creative ideas, while openness to experience (Silvia, 2008) correlates with the selection of more creative ideas. Information about the identity of the producer can also bias evaluators’ assessments: evaluators often rely on perceptions of the producer’s age, mental and emotional stability, and “genius” to guide their creativity evaluations (e.g., Baas et al., 2016; Rietzschel et al., 2019; Simonton, 2004, 2014). Judgments also depend on evaluators’ emotional states, whether positive, negative, or neutral (e.g., Mastria et al., 2019).

^3^For example, in studies conducted by Kruger et al. (2004), participants were exposed to the same poem (in experiment 1), paintings (in experiment 2), or arms and armor (in experiment 3), but were given different information about the time the artist spent writing the poem (four or 18 h), creating the painting (four or 26 h), or making the arms and armor (15 or 110 h). The findings revealed that participants gave more favorable evaluations of the artworks when they believed the creation process took longer. The influence of time on judgment was particularly evident when it was difficult for participants to evaluate the quality of the artwork itself (achieved in their study by using low versus high-resolution images).
